# Biophysical modeling and experimental validation of relative biological effectiveness (RBE) for ^4^He ion beam therapy

**DOI:** 10.1186/s13014-019-1295-z

**Published:** 2019-07-11

**Authors:** Stewart Mein, Ivana Dokic, Carmen Klein, Thomas Tessonnier, Till Tobias Böhlen, Guiseppe Magro, Julia Bauer, Alfredo Ferrari, Katia Parodi, Thomas Haberer, Jürgen Debus, Amir Abdollahi, Andrea Mairani

**Affiliations:** 1 Division of Molecular and Translational Radiation Oncology, Heidelberg University Medical School, Heidelberg Institute of Radiation Oncology (HIRO), National Center for Radiation Research in Oncology (NCRO), Heidelberg, Germany; 2 Heidelberg Ion-Beam Therapy Center (HIT), Department of Radiation Oncology, Heidelberg University Hospital (UKHD), Heidelberg, Germany; 3 Translational Radiation Oncology, German Cancer Consortium (DKTK) Core Center, German Cancer Research Center (DKFZ), Heidelberg, Germany; 4 National Center for Tumor Diseases (NCT), Heidelberg, Germany; 5 Heidelberg University, Faculty of Physics, Heidelberg, Germany; 6 Centre François Baclesse, Radiation Oncology, Medical Physics Department, Caen, France; 7 Center for Proton Therapy, Paul Scherrer Institute (PSI), Villigen, Switzerland; 8 National Centre of Oncological Hadrontherapy (CNAO), Medical Physics, Pavia, Italy; 9 European Organization for Nuclear Research (CERN), Geneva, Switzerland; 10 Ludwig-Maximilians-Universität (LUM Munich), Munich, Germany

**Keywords:** Particle therapy, Helium ions, Relative biological effectiveness, Translational research

## Abstract

**Background:**

Helium (^4^He) ion beam therapy provides favorable biophysical characteristics compared to currently administered particle therapies, i.e., reduced lateral scattering and enhanced biological damage to deep-seated tumors like heavier ions, while simultaneously lessened particle fragmentation in distal healthy tissues as observed with lighter protons. Despite these biophysical advantages, raster-scanning ^4^He ion therapy remains poorly explored e.g., clinical translational is hampered by the lack of reliable and robust estimation of physical and radiobiological uncertainties. Therefore, prior to the upcoming ^4^He ion therapy program at the Heidelberg Ion-beam Therapy Center (HIT), we aimed to characterize the biophysical phenomena of ^4^He ion beams and various aspects of the associated models for clinical integration.

**Methods:**

Characterization of biological effect for ^4^He ion beams was performed in both homogenous and patient-like treatment scenarios using innovative models for estimation of relative biological effectiveness (RBE) in silico and their experimental validation using clonogenic cell survival as the gold-standard surrogate. Towards translation of RBE models in patients, the first GPU-based treatment planning system (non-commercial) for raster-scanning ^4^He ion beams was devised in-house (FRoG).

**Results:**

Our data indicate clinically relevant uncertainty of ±5–10% across different model simulations, highlighting their distinct biological and computational methodologies. The *in vitro* surrogate for highly radio-resistant tissues presented large RBE variability and uncertainty within the clinical dose range.

**Conclusions:**

Existing phenomenological and mechanistic/biophysical models were successfully integrated and validated in both Monte Carlo and GPU-accelerated analytical platforms against *in vitro* experiments, and tested using pristine peaks and clinical fields in highly radio-resistant tissues where models exhibit the greatest RBE uncertainty. Together, these efforts mark an important step towards clinical translation of raster-scanning ^4^He ion beam therapy to the clinic.

**Electronic supplementary material:**

The online version of this article (10.1186/s13014-019-1295-z) contains supplementary material, which is available to authorized users.

## Background

With nearly 150,000 patients treated globally to date, particle therapy has revolutionized cancer therapy by offering enhanced precision and radiobiological properties over the conventional photons [1]. At the Heidelberg Ion-Beam Therapy Center (HIT), proton (^1^H) and carbon (^12^C) ion beams, the leading modalities in hadrontherapy, are applied clinically, with two additional particle species available for experimentation: oxygen (^16^O) and helium (^4^He) ion beams. Interest in medical applications using helium ions began during the clinical trials at Lawrence Berkeley Laboratory (LBL) between the years of 1977 and 1993, with over 2000 patients successfully treated [2]. Since the program’s end, ^4^He ion beams remain clinically unexploited.

It is well known that, experimentally, heavier ions exhibit greater biological damage and consequently, the biophysical properties of ^4^He are intermediate of the two clinically administered particle beams. That being said, application of helium ions provides a distinct clinical advantage, i.e. favorable dose distributions with attributes such as a sharper Bragg peak and lateral penumbra (reduced range straggling and scattering) compared to protons, and similar potential for tumor control with a substantially reduced fragmentation tail compared to carbons ions [3, 4]. With these characteristics, helium ions have been proposed as an ideal treatment option for radio-resistant diseases and delicate patient cases e.g. meningioma and pediatrics [5, 6].

Next year, HIT will launch the first European clinical program using therapeutic ^4^He ion beams, which marks the world’s first clinical application of raster-scanning ^4^He ion therapy. Over the past decade, substantial efforts have been made at HIT to characterize ^4^He ion beams via measurement and FLUKA Monte Carlo (MC) simulation [7, 8] both dosimetrically, i.e. in terms of depth and lateral dose distributions with single pencil beam (PB) and spread-out Bragg peak (SOBP) plans, as well as nuclear fragmentation [9–12]. In addition, classification of the beam’s biological effects is in progress, studying both in silico [5] and clonogenic cell survival in clinically-relevant conditions [13–15]. Presently, there is no commercial treatment planning system (TPS) available for ^4^He ion beams; however, research-based tools were recently introduced or updated to allow planning with ^4^He ion beams [10, 14, 16].

Relative to the clinical standard photons and protons, ^4^He ion beams exhibit, in certain cases, more advantageous biological dose distributions with a higher linear energy transfer (LET) [17] in the tumor, resulting in superior relative biological effectiveness (RBE) in the target compared to the entrance channel, a valuable attribute for treatment of deep-seated radio-resistant tumors. To anticipate variability of tissue-specific radio-sensitivity in the clinic, the TPS predictions of physical dose will be coupled with a biophysical (RBE) model for calculation of an effective dose.

In contrast with proton RBE with nearly 300 experimental *in vitro* measurements, data for helium is relatively scarce (~ 1/3 as large), leading to larger uncertainties in helium RBE. As for *in vivo* investigation of ^4^He ion beams, few publications examine evidence of enhanced tumor control compared to conventional techniques, most of which originate from the LBL trials from prior decades, yet only a fraction of these works relate findings to RBE [18, 19]. In preparation for the first patient treatment with ^4^He ion beams at HIT, we compared the predictions of three existing RBE models to biological measurements *in vitro* with monoenergetic beams and in clinically-relevant scenarios, as well as highlighting the inter- and intra-model variations as a function of tissue type, dose level, LET_d_, depth and beam configuration in silico. For the *in vitro* study, a cell line exhibiting substantial radio-resistance was selected for irradiation with both pristine beams and clinical-like fields. These more radio-resistant tissues (α/β < 4Gy) are of particular interest considering they make up only ~ 5% of the available experimental data in the literature for ^4^He ion beams. In addition to *in vitro* study, patient treatment plans were calculated and compared, applying the various ^4^He RBE schemes in place of a constant RBE [20]. The three published models for RBE prediction with ^4^He ion beams investigated in this study are as follows: a data-driven phenomenological model (DDM) [13, 14] and two biophysical models featuring the Local Effect Model (LEM, version IV) [21] and the modified Microdosimetric Kinetic Model (MKM) [22, 23]. With a long-term outlook in mind for ^4^He RBE study and clinical integration, this work can serve as a foundation for clinical decision-making regarding effective dose calculation, in preparation for the first ^4^He ion beam therapy patient treatments in Europe.

## Methods and materials

### Experimental investigations

#### Cell culture and clonogenic assay

Murine renal adenocarcinoma cells (Renca ATCC® CRL-2947™) were cultured in RPMI-1640 Medium (Gibco, Germany) supplemented with 10% heat-inactivated Fetal Bovine Serum (FBS, Millipore, Germany) and 1% Penicillin/Streptomycin (Gibco, Germany) at 37 °C and 5% CO_2_ atmosphere. Clonogenic cell survival assay, i.e. seeding, irradiation, incubation and read-out, was performed as previously described using 96-well plates [24]. Image acquisition took place with the IncuCyte® System (Essen BioScience, UK) for colony counting. A baseline characterization of the cell line was performed separately prior to *experiment A* (pristine peaks) and *experiment B* (SOBPs), which involved photon irradiation delivery (LINAC, 6 MV, Artist Siemens) with dose levels of 1, 2, 4, and 8 Gy for determination of the LQ parameters (α_x_ and β_x_).

#### Irradiation with Monoenergetic beams

To most closely resemble track segment conditions, cell were irradiated with monoenergetic ^4^He beams (E_4-He_ = 56.66 MeV/u, d_BP_ = 25.9 mm) in *experiment A*. Two sets of biological measurement points were taken at 6 mm and 12 mm water-equivalent depth (WED). Cell-kill measurements were collected for the pristine beams at dose levels of approximately 0.25, 0.5, 0.75, 1.0, 1.5, 2.0, 2.5, and 3.0 Gy. Dosimetric measurements were performed using a Farmer ionization chamber (TM30010, PTW, Freiburg) for validation of FLUKA MC predictions.

#### Irradiation with SOBPs

**Fig. 1:**
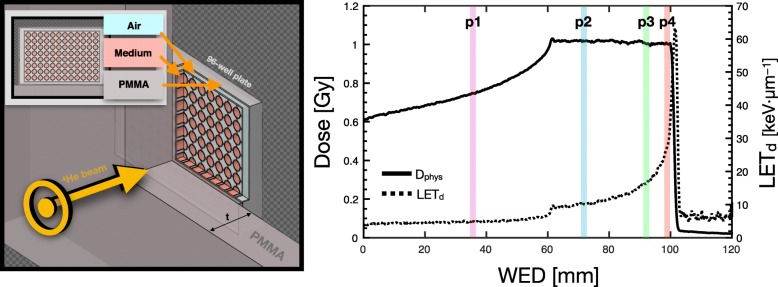
Left: cross-section of schematic for the 96-well plate geometry and composition in FLUKA MC for *experiment A* and *B*. Right: central line profile through physically optimized SOBP plan for *experiment B*, displaying both physical dose and LET_d_. The biological measurement positions are designated by the highlighted regions (p1, p2, p3, p4)

For investigating clinical-like conditions, the same plate configuration was used as in the base-line photon irradiations. *Experiment A* and *B* involved 96-well plates positioned against various thicknesses of PMMA, such that each plate corresponded to a specific depth (and hence, LET_d_) in the SOBP irradiation [24], with positions of 3.0 cm (p1), 5.98 cm (p2), 7.61 cm (p3), and 8.35 cm (p4) in PMMA. WED values were calculated using a multiplicative factor of 1.165 and are highlighted in Fig. 1 (right panel). SOBP plans were physically optimized in water for the following doses in the 12 cm × 8 cm × 4 cm target region centered at 8 cm depth: 0.5, 1.0, 2.0, 3.0, 4.0 and 6.0 Gy. The 96-well plate geometry with corresponding material composition was integrated into the FLUKA MC simulation.

**Table 1:** Photon parameters applied during the in silico investigations. The D_t_ parameter is required for LEM calculations only

α_x_ [Gy^−1^]	β_x_ [Gy^− 2^]	(α/β)_x_ [Gy]	D_t_ [Gy]	calculation type	References
0.2	0.1	2	6.2	water	
0.2	0.02	10	15	water	
0.036	0.024	1.5	5.65	prostate	Brenner and Hall (1999) [39]
0.089	0.0287	3.1	7.41	prostate	Terry and Denekamp (1984) [41]
0.077	0.009	8.6	13.41	head	Jones and Sanghera (2007) [42]
0.0499	0.0238	2.1	6.31	head	Meeks et al. (2000) [43]

### Models and MC simulation

#### Modeling the relative biological effectiveness of ^4^He ion beams

Biological dose prediction begins with modeling cell survival (S), traditionally described as a linear-quadratic (LQ) trend, with α and β representing the linear and quadratic coefficients, respectively, as a function of physical dose (D). The ratio of the linear and quadratic coefficients, (α/β)_x_, is often referred to as a description for the sensitivity of the cell line when exposed to photon radiation (x). The RBE is a multifunctional quantity defined as the isoeffective dose ratio between a reference radiation (D_x_) and a particle radiation (D_p_), traditionally modeled as a function of three parameters: (α/β)_x_, LET and D_x_. Biological (or effective) dose (D_RBE_) is defined as the product of the RBE and the physical dose.

Within the LQ framework, we can determine a dependency of RBE on (α/β)_x_, the helium absorbed dose, RBE_α_ and R_β_ [13, 14]:

2 $$ \mathrm{RBE}\left({\left(\frac{\upalpha}{\upbeta}\right)}_{\mathrm{x}},\mathrm{D},{\mathrm{R}\mathrm{BE}}_{\upalpha},{\mathrm{R}}_{\upbeta}\right)=-\frac{1}{2\mathrm{D}}{\left(\frac{\upalpha}{\upbeta}\right)}_{\mathrm{x}}+\frac{1}{\mathrm{D}}\sqrt{\frac{1}{4}{\left(\frac{\upalpha}{\upbeta}\right)}_{\mathrm{x}}^2+{\mathrm{R}\mathrm{BE}}_{\upalpha}{\left(\frac{\upalpha}{\upbeta}\right)}_{\mathrm{x}}\mathrm{D}+{\mathrm{R}}_{\upbeta}{\mathrm{D}}^2} $$

In the next sections, the expressions for RBE_α_ and R_β_ per the three models will be introduced. In the case of the LEM, the LQ approximation for the photon response is valid up to threshold dose D_t_, which marks the transition dose at which the survival curve for photon irradiation is assumed to have an exponential shape with the maximum slope S_max_ = α_x_ + 2β_x_D_t_ [25]. In this work, the dose levels have been chosen within the range of LQ applicability, i.e. < D_t_.

The predictions of the three RBE models have been assessed by comparing RBE_α_ and R_β_ as a function of LET, and the RBE values as a function of LET and dose for two tissue types irradiated with ^4^He ion beams. Parameters characterizing the hypothetical tissues considered for this study are reported in Table 1 and labeled water case. The (α/β)_x_ values were selected similar to recent works [26] to represent late-responding tissues (low (α/β)_x_ from 2 to 3 Gy), and early-responding normal tissues and most common tumors (high (α/β)_x_ from around 10 Gy).

**Table 2:** Clonogenic cell survival LQ fit parameters for photon (α_x_ and β_x_) and helium ion beam (α and β) irradiation using the Renca cells *in vitro* with corresponding LET_d_ derived from MC simulation. Data for both *experiment A* (pristine peaks) and *experiment B* (SOBPs) are provided

Exp.	α_x_ [Gy^−1^]	β_x_ [Gy^−2^]	(α/β)_x_ [Gy]	α [Gy^−1^]	β [Gy^−2^]	LET_d_ [keV/μm]
A	0.034 (±0.004)	0.018 (±0.001)	1.79	0.039 (±0.013)	0.029 (±0.003)	5.33
A	0.034 (±0.004)	0.018 (±0.001)	1.79	0.094 (±0.012)	0.046 (±0.012)	14.81
B	0.050 (±0.064)	0.023 (±0.014)	2.17	0.076 (±0.083)	0.024 (±0.02)	4.78
B	0.050 (±0.064)	0.023 (±0.014)	2.17	0.150 (±0.071)	0.018 (±0.018)	10.18
B	0.050 (±0.064)	0.023 (±0.014)	2.17	0.201 (±0.048)	0.017 (±0.005)	15.37
B	0.050 (±0.064)	0.023 (±0.014)	2.17	0.305 (±0.144)	0.022 (±0.032)	26.52

#### Data-driven LET-based model

A phenomenological model for RBE with ^4^He ion beams was developed by fitting *in vitro* experimental data available in the literature in Mairani et al. 2016a [11] and refined in Mairani et al. 2016b [12]. For RBE_α_, the following parameterization has been introduced:

3 $$ {\mathrm{RBE}}_{\upalpha}=1+\left[{\mathrm{k}}_0+{\left(\frac{\upalpha}{\upbeta}\right)}_{\mathrm{x}}^{-1}\right]{\mathrm{k}}_1{\mathrm{L}}^{\ast }{\mathrm{e}}^{-{\mathrm{k}}_2{\mathrm{L}}^{\ast 2}} $$

where L* represents the rescaled ^4^He LET [13]:

4 $$ {\mathrm{L}}^{\ast }=\mathrm{LET}-{\mathrm{L}\mathrm{ET}}_{\mathrm{x}}+{\mathrm{L}\mathrm{ET}}_{{}{}^{60}\mathrm{Co}} $$

LET_x_ and ${\mathrm{LET}}_{{}{}^{60}\mathrm{Co}}$ are, respectively, the LET of photon under study and of the reference ^60^Co. The parameters used in eq. 3 are as follows [12]: k_0_ = 8.924 × 10^− 2^ Gy^− 1^ and k_1_ = 3.368 × 10^− 1^ μm**·**keV^−1^, and k_2_ = 2.858 × 10^− 5^ μm^2^**·**keV^− 2^. For R_β_, we have introduced an LET-dependent parameterization fitting the running averages of R_β_ as function of LET:

5 $$ {\mathrm{R}}_{\upbeta}={\mathrm{b}}_0{\mathrm{e}}^{\left[-{\left(\frac{{\mathrm{L}}^{\ast }-{\mathrm{b}}_1}{{\mathrm{b}}_2}\right)}^2\right]} $$

The coefficients for the R_β_ parameterization are b_0_ = 2.66, b_1_ = 62.61 keV μm^−1^ and b_2_ = 48.12 keV μm^−1^ .

For comparison in track-segment conditions, we have assumed L^*^ = LET while for the clinically-relevant scenarios and *in vitro* studies, we used 6 MV photon beams as a reference radiation for calculating rescaled L^*^ values.

#### Modified Microdosimetric kinetic model (MKM)

In the modified MKM [22, 23], for any radiation quality, RBE_α_ is expressed as a function of the saturation-corrected dose-mean specific energy of the domain delivered in a single event ${\mathrm{z}}_{1\mathrm{D}}^{\ast }$ divided by the (α/β)_x_ ratio:

6 $$ {\mathrm{RBE}}_{\upalpha}=1+{\left(\frac{\upalpha}{\upbeta}\right)}_{\mathrm{x}}^{-1}\bullet {\mathrm{z}}_{1\mathrm{D}}^{\ast } $$

${\mathrm{z}}_{1\mathrm{D}}^{\ast }$ depends on *z*, the specific energy, and *z*_*sat*_, the saturation-corrected specific energy which accounts for the decrease of RBE due to the overkilling effect for high specific energy values [27]. *z* depends on the radius of the domain (*R*_*d*_) while *z*_*sat*_ depends *R*_*d*_ and the radius of the cell nucleus (*R*_*n*_) [22]. MKM input parameters (*R*_*d*_ and *R*_*n*_) have been tuned in a previous work [22] to reproduce an *in vitro* experimental biological database of initial RBE. The resulting best fit values of *R*_*d*_ = 0.3 *μm* and *R*_*n*_ = 3.6 *μm* obtained in Mairani et al. 2017 have been used in this work without further adjustments. For the R_β_ term, it is assumed [28]:

7 $$ {\mathrm{R}}_{\upbeta}=1. $$

#### Local effect model (LEM)

The LEM-version IV developed by the GSI Helmholtz Centre for Heavy Ion Research (Darmstadt, Germany) [21] relates the biological response directly to the double-strand breaks pattern and has been benchmarked by its developers in various publications [10, 21]. The LEM intrinsic α_z_ tables are obtained using the PT RBE Generator software by Siemens which is available at HIT, while for β_z_, we have used the approximation β_*z*_ = (*s*_*max*_ − *α*_*z*_)/(2*D*_*t*_), with negative values found at high LET forced to zero [25]. The LQ parameters are calculated at different energies applying the low dose approximation, which describes how to link the input LEM-calculated intrinsic microscopic parameters, α_z_ and β_z_, to the macroscopic values, α and β. The initial RBE can be written as:

8 $$ {\mathrm{RBE}}_{\upalpha}=\frac{1-{\mathrm{e}}^{-{\upalpha}_z{\mathrm{d}}_1}}{\upalpha_x{\mathrm{d}}_1} $$

with R_β_ as:

9 $$ {\mathrm{R}}_{\upbeta}={\left(\frac{\upalpha}{\upalpha_z}\right)}^2\left(\frac{\upbeta_z}{\upbeta_x}\right) $$

d_1_ is the dose deposited by a single particle traversal [29, 30].

#### MC simulation of the *in vitro* study

For both *experiment A* and *B*, the target (96-well plate irradiation system) was incorporated into FLUKA MC, including a detailed geometry of the HIT beam-line [31], for validating the biological dose models against experimental measurements. Once biological measurements were acquired, simulations were executed to score physical dose and LET_d_, as well as the various biological parameters necessary for D_RBE_ using the DDM, MKM and LEM. With a detailed geometry of the 96-well plate target, parameters were scored on a per well basis to reduce physical and biological uncertainties during evaluation of measurement and simulation outcomes, as shown in Fig. 1. Cell survival and, in turn, RBE results were compared to MC prediction to validate enhanced cell-kill with increased LET_d_ for helium ions and to evaluate model performance.

### Patient studies and validations

#### Retrospective study: patient treatment planning and forward computation of D_RBE_

In this work, the MC-based treatment planning tool (MCTP) [32, 33] is employed to create biologically optimized treatment plans and to perform forward dose calculation for retrospective study. The MCTP relies on FLUKA’s capability to describe the interaction and transport of radiation with matter for ^4^He ion beams and is coupled with both biophysical and phenomenological RBE models for ^4^He. FLUKA has been benchmarked against dosimetric data, demonstrating overall a satisfactory agreement [11].

The MCTP uses dosimetrically commissioned scanned pencil beams as available at HIT [34]. The data-driven RBE model has been used for treatment plan optimization. The MCTP tool relies on externally generated databases for each biological effect model to calculate RBE and D_RBE_ values [37, 38]. To properly calculate effective dose for helium ion beams, Z = 2 primary particles and secondary fragments as well as Z = 1 secondary fragments must be scored separately. Hence, both the DDM and a phenomenological model for Z = 1 were used during biological dose weighting of Z = 2 and Z = 1, respectively [35].

MCTP-based plans have been calculated to achieve a homogeneous three-dimensional D_RBE_ of 2.0 Gy (RBE) and 4.0 Gy (RBE) in the target region with a single field and a two opposing fields arrangement in water. Two targets were chosen: rectangular parallelepiped volumes of 6 cm × 6 cm × 6 cm and 3 cm × 3 cm × 3 cm centered at 12.5 cm water-equivalent depth. FLUKA MC scoring for physical and biological quantities was performed in voxels of 2 mm × 2 mm × 2 mm. The lateral PB spacing was 3 mm while the depth separation between Bragg peak positions of two consecutive energy slices was 2 mm. The plans have been calculated assuming two representative tissues with (α/β)_x_ of 2 Gy and 10 Gy as reported in the first two rows of Table 1.

MCTP-based plans for two patients (previously treated with protons at HIT) were simulated using one and two ^4^He ion beam portals. Beam configurations for the head and prostate case involved a single field (superior-inferior direction) and parallel opposed fields (anterior-posterior / posterior-anterior direction), respectively. To achieve dose homogeneity in the target of the head case, a ripple filter has been used for broadening the beam longitudinally [36]. The lateral PB spacing was 3 mm while depth separation between two consecutive energy slices was 3 mm. FLUKA MC scoring was performed in voxels of 1 mm × 1 mm × 3 mm. The planned doses were 54 Gy (RBE) in 27 fractions and 66 Gy (RBE) in 20 fractions for the head and the prostate cases, respectively [35], applying the clincal fractionation scheme used at HIT with proton beams.

Forward re-computation of the optimized plans have been carried out to investigate the variation of the D_RBE_ as a function of the depth, applying the biophysical models previously described. LET_d_ distributions were additionally scored for dose values larger than 5% of the maximum *Z* = 2 dose. For dose distribution characterization in the target of the SOBPs, equivalent uniform dose (EUD) was applied [37]. We have calculated EUD as follows [38]:

10 $$ \mathrm{EUD}=-\frac{1}{2}{\left(\frac{\upalpha}{\upbeta}\right)}_{\mathrm{x}}+\sqrt{\frac{1}{4}{\left(\frac{\upalpha}{\upbeta}\right)}_{\mathrm{x}}^2-\frac{\ln \left(\overline{\mathrm{S}}\right)}{\upbeta_{\mathrm{x}}}} $$

where $\overline{\mathrm{S}}$ is the mean survival in the target. For the patient cases, we have also analyzed the D_RBE_ volume histograms (D_RBE_VH).

Following the previous works [14], tissue parameters for the prostate case were set to α_x_ = 0.036 Gy^−1^ and β_x_ = 0.024 Gy^−2^ for (α/β)_x_ = 1.5 Gy [39, 40]. For the surrounding healthy tissues, (α/β)_x_ = 3.1 Gy with α_x_ = 0.089 Gy^−1^ and β_x_ = 0.0287 Gy^−2^ was applied [41]. For the head patient case, for the planning target volume (PTV) assuming a glioma tumor, we have used α_x_ = 0.077 Gy^−1^ and β_x_ = 0.009 Gy^−2^, yielding (α/β)_x_ = 8.6 Gy [42] while for the rest of the brain, we have assumed α_x_ = 0.0499 Gy^−1^ and β_x_ = 0.0238 Gy^−2^, yielding (α/β)_x_ = 2.1 Gy [43]. Further details regarding these values are provided in Table 1.

### Development and validation of an analytical biological dose calculation engine: FRoG

Once patient case dose calculation was established via biological dose models coupled with FLUKA, validation of the fast (GPU-based) analytical dose engine, FRoG, was performed [44, 45]. Physical and biological parameter database generation took place using FLUKA MC simulation. Corresponding biological parameters for DDM (α_He_ and β_He_), LEM (α_He_ and β_He_), and MKM (${\mathrm{z}}_{1\mathrm{D}}^{\ast }$) were scored as a function of depth, along with the necessary physical parameters (dose and LET_d_). The physical and biological tables were incorporated into the FRoG platform, enabling multi-tissue (variable (α/β)_x_) dose calculation for the three biological dose models. The glioma patient plan was executed in FRoG for comparison with the gold standard FLUKA MC.

All patients records were anonymized prior to the study, obtained with informed consent and handled following the Helsinki Declaration. All methods were approved by the Heidelberg University Medical Faculty, following applicable guidelines and regulations of the institution.

## Results

### Investigating model dependencies in silico: SOBPs and patient cases

**Fig. 2:**
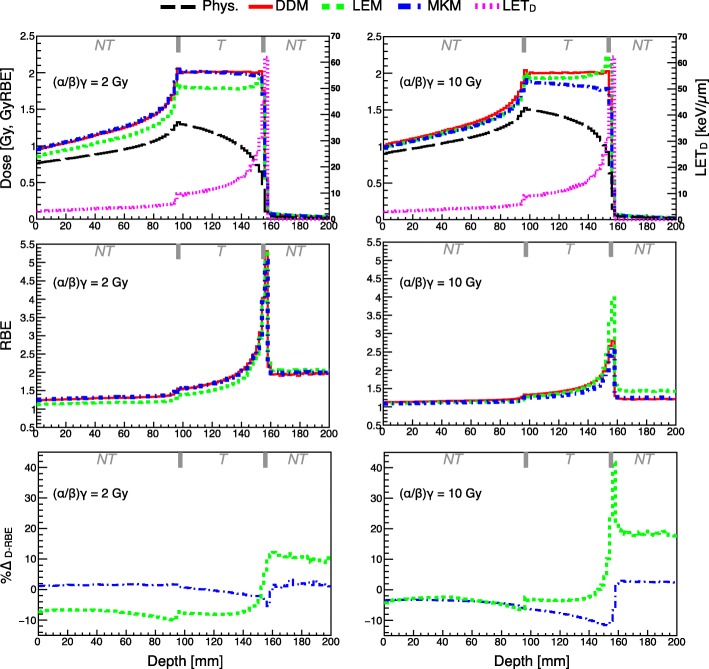
Biologically optimized SOBPs at 2 Gy (RBE) using the data-driven model (DDM) assuming two distinctive tissue types with (α/β)_x_ = 2 Gy (left) and (α/β)_x_ = 10 Gy (right) are displayed as function of the depth in water, plotted against forward calculations with the two biophysical models applied, as well physical dose and LET_d_ distributions (calculated via MC simulation). RBE variation as a function of depth with percent difference D_RBE_ (%∆_D-RBE_) between planned and forward calculation D_RBE_ for MKM and LEM are displayed in the middle and bottom panel, respectively. The top axis of each figure segments regions of normal tissue (NT) and tumor tissue (T) for a representative clinical treatment

Clinically relevant scenarios were used to further characterize model variations. Figure 2 presents RBE-weighted dose (D_RBE_) for the SOBPs, calculated via MC simulation, as a function of depth in water for the three investigated models, as well as physical dose and LET_d_**.** RBE variation and %∆_RBE_ are also visualized in the following middle and lower panels, respectively. The SOBP plan, biologically optimized using the DDM, was applied to reach a biological dose level of 2 Gy (RBE) and for two tissue types, exhibiting (α/β)_x_ of 2 Gy and 10 Gy, experimental surrogates for testing radio-resistant and radio-sensitive tissues, respectively. A similar investigation was executed for an irradiation plan with two opposing fields, as shown in Additional file 1: Figure S1. For quantification of global difference in the target between the various models, EUD calculations for the SOBPs studied in silico are provided in Additional file 1: Table S2 and S3.

### Model dependencies in clinically-relevant scenarios: patient cases

**Fig. 3:**
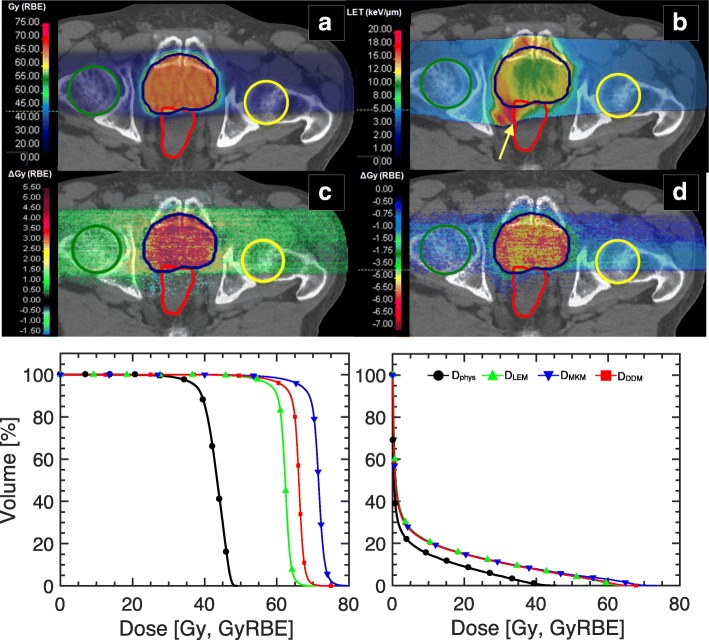
D_RBE_ comparison illustrated in a clinical case (prostate cancer). **a** MC-optimized D_RBE_ distribution applying the DDM for the prostate case with resulting (**b**) LET_d_ distribution for Z = 2 particles. **c** Differences between the reference D_RBE_ (DDM) and LEM and MKM D_RBE_ are displayed in panels (**c**) and (**d**), respectively. Contours for the PTV, femurs and rectum are displayed in blue, green/yellow and red, respectively. DVH and D_RBE_VH for the three biological models are depicted for the PTV and the rectum in the bottom left and right panel, respectively. Note that the critical organs at risk (e.g. anterior rectum) are susceptible to large variations in small volumes (< 5% of the relative total volume per organ) due to overlap with the tumor structure delineation. The asymmetric LET_d_ gradient (indicated by the yellow arrow) in panel (**b**) is indicative of the applied beam optimization procedure to meet dose constraints in the rectum while maintaining target coverage

In Fig. 3, an investigation of RBE model performance of a prostate cancer patient in silico is displayed. The MCTP calculated D_RBE_ distribution for the pelvic case applying the DDM and LET_d_ distribution are shown as well as dose difference (∆_Gy(RBE)_) from the reference when performing forward calculations with LEM and MKM. The physical dose volume histogram (DVH) and biological dose volume histogram (D_RBE_VH) for the PTV and rectum, chosen as a representative organ at risk (OAR), are displayed in the bottom panel. DVH statistics for the PTV in terms of D_50%_, D_RBE-50%_ and the inhomogeneity coefficient I_5%_ = (D_5%_ - D_95%_) / D_RBE,p_ have been analyzed. D_RBE-50%_, D_RBE-5%_, and D_RBE-95%_ represent the biological dose received by 50%, 5% and 95% of the PTV volume in the cumulative D_RBE_VH, respectively. D_RBE,p_ is the prescribed biological dose. I_5%_ evaluates the biological dose gradient introduced in the PTV by performing forward calculation of the patient plans with the various RBE models. LEM resulted in −5.7% lower D_50%_, while applying the MKM yielded 8.3% higher D_50%_. The I_5%_ values were, respectively, ~ 12% for MKM, and ~ 10% for both LEM and the reference (D_RBE_ calculated with DDM). The D_5%_ for the rectum was 50.2 Gy (RBE) for MKM, 46.0 Gy (RBE) for LEM and 48.2 Gy (RBE) for DDM.

### Validating RBE models in a clinical platform: FRoG

**Fig. 4:**
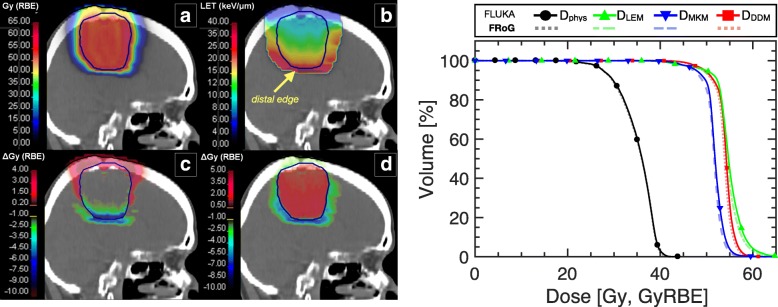
Validation of the FRoG dose engine for helium ion beam therapy dose calculation with a glioma patient case. D_RBE_ applying (**a**) DDM and (**b**) LET_d_ is displayed, along with dose difference between D_RBE_ applying DDM and (**c**) LEM and (**d**) MKM. DVH and D_RBE_VH for the three biological models are depicted for the PTV (right panel) for FRoG versus FLUKA. The yellow arrow directs attention to the LET_d_ gradient at the distal edge of the tumor, which could lead to larger uncertainty in RBE prediction for both the tumor and neighboring heathy issues beyond the target

A glioma patient case is displayed in Fig. 4 for RBE evaluation and validation of a fast analytical dose calculation engine (FRoG). FRoG calculation run-time for the glioma patient (yielding D and D_RBE_ applying DDM, MKM and LEM) was 142 s, a time gain factor of ~ 225 when compared to MC simulation using a 300 node CPU-cluster. The MCTP calculated D_RBE_ distribution for the head case applying the DDM and the resulting LET_d_ distribution are shown as well as dose difference ∆_Gy (RBE)_ from the reference when performing forward calculations with (c) LEM and (d) MKM. For the LEM- and MKM-based forward biological dose calculations, D_50%_ for the PTV is 1.5% higher and −3.7% lower, respectively, than the reference. Larger I_5%_ values were found for LEM and MKM of ~18% and ~14%, respectively, relative to the reference of ~13%. The greatest variations between the models occur for the normal tissue with (α/β)_x_ = 3.1 Gy, outside of the PTV, especially in the distal region where the highest LET components of the distribution are prevalent. For the glioma patient case, there are no OARs in proximity of the target.

As shown in Fig. 4, DVH and D_RBE_VH plots between FRoG and FLUKA are in good agreement. The percent absolute deviations in D_50%_ and D_RBE-50%_ for the PTV between FLUKA and FRoG for physical dose (D_phys_) and the three biological doses are as follows: 0.2, 0.4, 0.4, 0.6%, for D_phys_, D_DDM_, D_LEM_ and D_MKM_, respectively. Further details regarding DVH and D_RBE_VH statistics are provided in Additional file 1: Table S1.

### Experimental evaluation of the RBE models

**Fig. 5:**
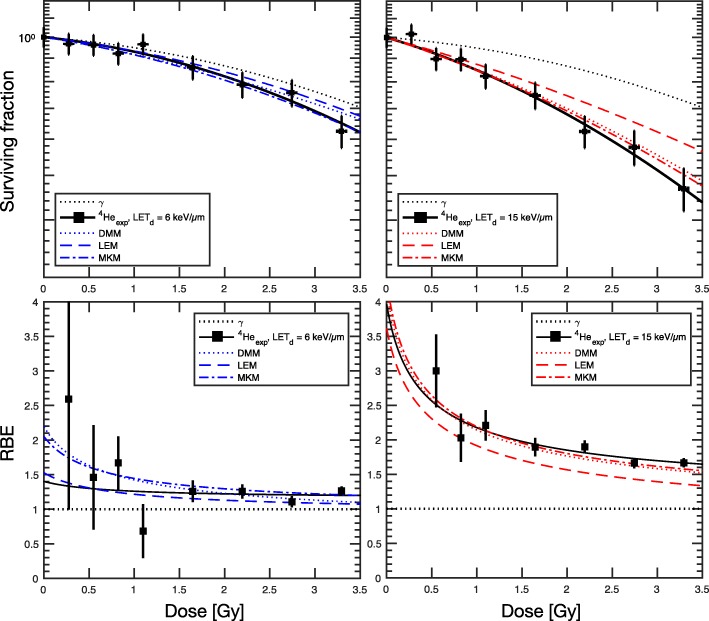
Clonogenic survival (top) of the Renca cells when exposed to various doses of a monoenergetic ^4^He ion beam at two measurements points. MC-estimated LET_d_ values are ~ 6 keV·μm^−1^ at 6 mm depth (upper left) and ~ 15 keV·μm^−1^ at 12 mm depth (upper right) using a ^4^He beam energy E = 56.65 MeV/u with a BP position (d_BP_) of 25.9 mm. FLUKA-coupled biophysical and phenomenological models predicted cell survival and corresponding RBE (bottom) with varying degree of accuracy as a function of dose. The dotted and solid black line represent the LQ-fit of the Renca cells photon irradiation (γ) with (α/β)_x_ = 1.79 Gy and ^4^He irradiation, respectively

Enhanced cell-killing was observed in the biological measurements of *experiment A* for higher LET_d_ (~ 15 keV·μm^− 1^) compared to lower LET_d_ (~ 6 keV·μm^− 1^). Figure 5 displays both the experimental findings (points with error bars) and FLUKA MC-coupled RBE model predictions for cell survival and RBE, as well as percent difference in RBE (%∆_RBE_) of the three models against experimental data. Linear quadratic (LQ) fitting of the cell survival data from photon irradiations with the 6MV LINAC yielded α_x_ = 0.034 Gy^− 1^ and β_x_ = 0.018 Gy^− 2^, for an (α/β)_x_ of 1.79 Gy. For the lower LET_d_ condition, LEM exhibited the most stable prediction of RBE as a function of dose below 1.5 Gy with %∆_RBE_ < 5% but consistently underestimates RBE. On the other hand, DDM and MKM yielded better RBE predictions from 1.5 Gy and above. For the higher LET_d_ condition, DDM and MKM predicted with the highest relative accuracy within the studied dose range, with %∆_RBE_ < 5% up to 2 Gy. LQ-fit parameters for two LET_d_ conditions are listed in the Table 2.

**Fig. 6:**
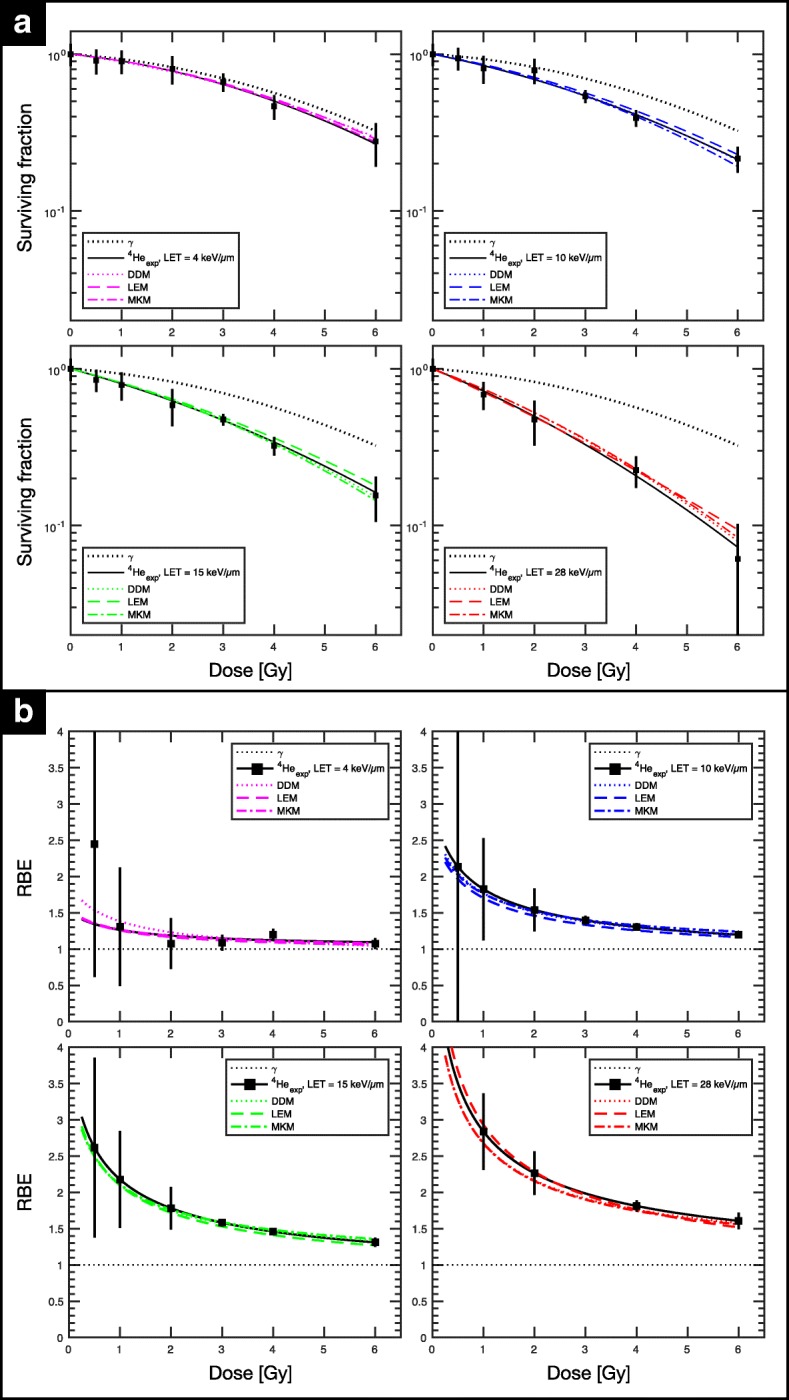
Clonogenic assay for clinical-like fields (SOBPs) for the Renca cell line in *experiment B*. MC simulation estimated LET_d_ values of biological measurement were ~ 5 keV·μm^−1^, ~ 10 keV·μm^−1^, ~ 15 keV·μm^−1^, ~ 27 keV·μm^−1^. FLUKA-coupled biophysical and phenomenological models predicted cell survival (**a**) and corresponding RBE **(b)** with varying degree of accuracy as a function of dose. The dotted and solid black line represent the LQ-fit of the Renca cells photon irradiation and ^4^He irradiation, respectively. LQ-fit parameters for the four LET_d_ conditions are listed in the Table 2

Regarding outcome of *experiment B*, initial investigation of cell-kill response to photon irradiation yielded α_x_ = 0.050 Gy^− 1^ and β_x_ = 0.023 Gy^− 2^, for an (α/β)_x_ of 2.17 Gy, which is on average 0.38 Gy higher than the (α/β)_x_ found in *experiment A*. Figure 6.a displays the cell survival versus dose for the four LET_d_ conditions (~ 5 keV·μm^− 1^, ~ 10 keV·μm^− 1^, ~ 15 keV·μm^− 1^, ~ 27 keV·μm^− 1^) within a clinically relevant dose range (D_phys_ ≲3 Gy). For both model predictions and experimental data, a dose dependence in RBE was observed in all cases. In general, DDM and MKM performed best for both higher and lower LET_d_ conditions in the studied dose range, consistent with findings from the monoenergetic beam experiment. RBE predictions for all three models agreed within ±5% of the experimental data for the two highest LET_d_ conditions (~ 15 keV·μm^− 1^ and ~ 27 keV·μm^− 1^), especially DDM and MKM for dose levels > 2 Gy. For 2 Gy, %∆_RBE_ for the four LET_d_ conditions (in ascending order) were roughly, + 3.7%, − 1.9%, − 1.9%, − 4.4% for DDM, − 1.7%, − 5.3%, − 3.4% and + 0.9% for LEM, and − 4.1%, − 1.1%, − 1.1% and − 4.8% for MKM. For the lower LET condition of ~ 5 keV·μm^− 1^ (entrance channel measurement), all models produced RBE predictions within ±5−10%, reaching ~ 1.3 for 0.5Gy, ~ 1.25 for 1 Gy, ~ 1.18 at 2 Gy and stabilizing to ~ 1.1 for the higher doses. As for the LET_d_ conditions found in the target (~ 10 keV·μm^− 1^, ~ 15 keV·μm^− 1^, ~ 27 keV·μm^− 1^), representing a low, mid and high range LET_d_ for therapeutic helium ion beams, respectively, greater variability was observed as a function of dose, especially for doses < 2 Gy. For 1 Gy, observed RBE values were ~ 1.8, ~ 2.2, ~ 2.8 for the low, mid and high LET_d_ conditions in the target. At 4 Gy, RBE values decreased to ~ 1.3, ~ 1.5, ~ 1.8 for the low, mid and high LET_d_ conditions.

## Discussion

### RBE model assessment

To best interpret the biological models for ^4^He ion beams, one must begin with a survey of their dependencies in track-segment conditions, i.e. monoenergetic beam case disregarding contributions from a mixed radiation field. In track-segment conditions, one can clearly discern the basis of intra- and intermodal variation as a function of dose, LET and tissue type.

**Fig. 7:**
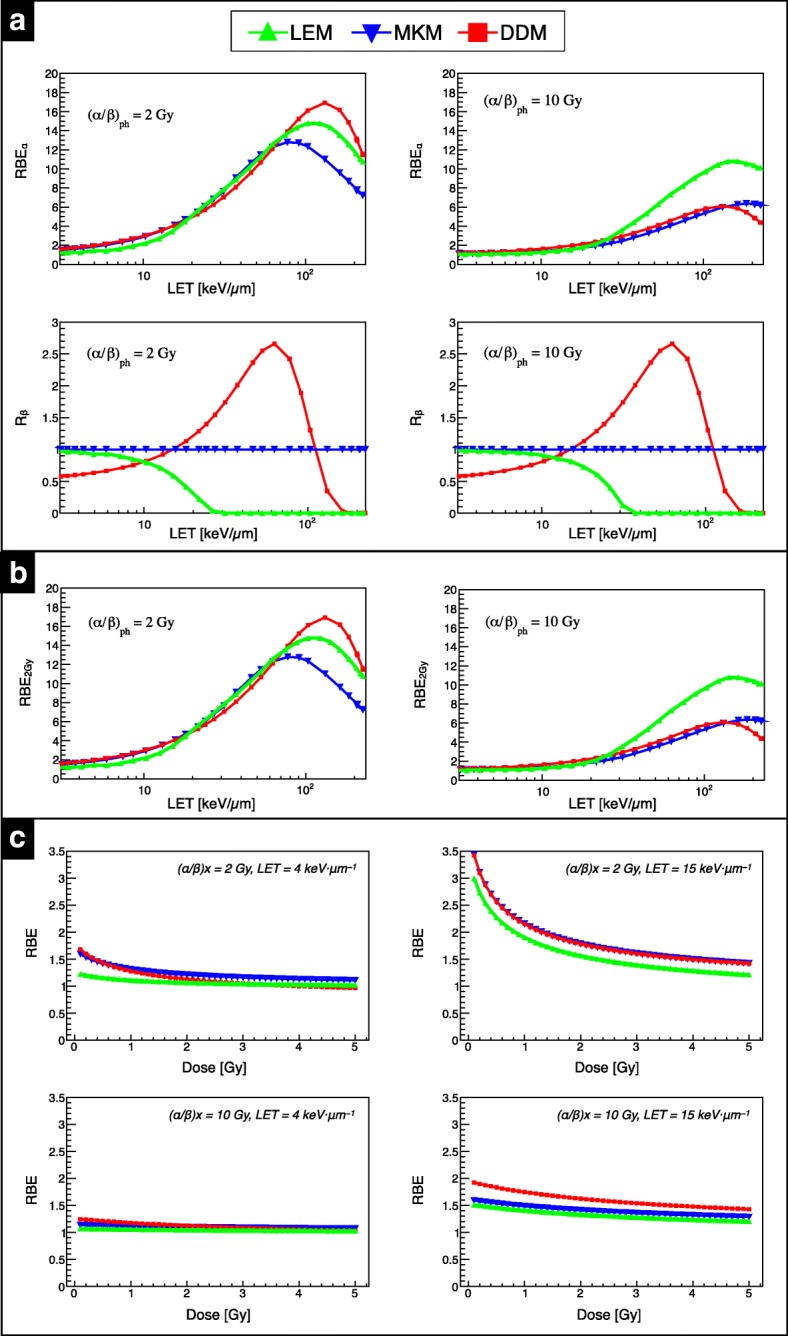
Comparison between the three model predictions. (**a**) RBE_α_ (top) and R_β_ (bottom) as function of LET for (α/β)_x_ = 2 Gy (left) and 10 Gy (right). (**b**) RBE as a function of LET for (α/β)_x_ = 2 Gy (left panel) and 10 Gy (right panel) at 2 Gy reference photon dose. (**c**) RBE as a function of ^4^He ion beam physical dose for (α/β)_x_ = 2 Gy (top) and 10 Gy (bottom) at 4 keV·μm^−1^ and 15 keV·μm^−1^ as shown in the left and right column, respectively

Figure 7.a shows the comparison of RBE_α_ (top) and R_β_ (bottom), for mono-energetic ^4^He ion beams as a function of LET for two tissues, (α/β)_x_ = 2 Gy (left panels) and 10 Gy (right panels), representing two distinct tissue types with differing responses to radiation. Comparison of these cases shows RBE_α_ and (α/β)_x_ are negatively correlated. As particle LET increases, an upward trend for RBE_α_ as a function of LET is observed, until a saturation point, where the RBE_α_ plateaus prior to fall-off. In general, this fall-off is more prominent and occurs at a lower LET range in lower (α/β)_x_ tissues. For lower LET, the largest inter-model variation occurs for the (α/β)_x_ = 2 Gy case between LEM and the other two models, while for the higher LET region, all models exhibit a varying response. For (α/β)_x_ = 10 Gy, the models yield similar predictions for LET values lower than about 20 keV·μm^− 1^. The location of RBE_α_ maximum changes as a function of the model applied.

Regarding R_β_, the models assume or predict different behaviors as function of LET. In the MKM [28], R_β_ is assumed to be unity, i.e. β_He_ = β_x_, while for the single-hit based version of LEM applied in this work [21], R_β_ decreases as LET increases. In the LET-based DDM approach, R_β_ increases with LET until reaching a maximum at ~ 63 keV·μm^− 1^ and then drops to zero for LET > 100 keV·μm^− 1^. For the data-driven approach, R_β_ is independent of (α/β)_x_, and therefore it’s behavior is consistent between tissue types. These differences in expressing R_β_ lead to significant variations among the models which, in part, reflect the large experimental uncertainties of the available experimental *in vitro* data [13].

RBE versus LET for the two tissues at physical dose levels of 2 Gy (left column) and 4 Gy (right column) are depicted in Fig. 7 b. As expected, the RBE initially increases with LET, reaches a maximum and then decreases. The RBE decreases for increasing dose mainly for low (α/β)_x_, and increases for decreasing (α/β)_x_ of the tissue. RBE results at lower LET and higher LET are presented as a function of physical dose for the two tissues. The chosen LET values are representative of the LET_d_ values found in the entrance channel and in the middle of an SOBP, respectively, for the two opposing beam fields arrangement depicted in Additional file 1: Figure S1. For clinical targets like an SOBP, one must consider a mixed radiation field with a complex LET spectrum, rather than a single LET value as in the case of an ion in the track-segment condition.

As expected, an enhanced RBE is observed at lower doses for all models, and this trend is more pronounced for lower (α/β)_x_ tissues. For the low LET condition, LEM predicted a limited RBE variation within the studied dose level, between maximum and minimum values, of about 20% and of about 4% for (α/β)_x_ = 2 Gy and (α/β)_x_ = 10 Gy, respectively. For 15 keV·μm^−1^ and for (α/β)_x_ = 2 Gy, MKM and the DDM approach resulted in roughly the same predictions, while for (α/β)_x_ = 10 Gy the DDM estimated about 15% higher RBE. In order to reduce model-related uncertainties in the target region, assuming 15 keV·μm^−1^ is a representative LET_d_ value for Z = 2 in the target, one could use hypo-fractionated treatments (D_RBE_ > 4 Gy (RBE)) where variations in RBE prediction decrease. In addition, hypo-fractionated treatments reduce the impact of precise (α/β)_x_ value assignment for target tissues on RBE determination. On the other hand, hypo-fractionation may diminish the therapeutic window by reducing the ratio of the target RBE compared to the entrance channel (i.e. tumor to normal tissue effective dose ratio). With typical peak-to-plateau dose ratio of ~ 2 for ^4^He ion beams and assuming a dose value of 4 Gy in target, RBE predictions (averaging over the three models in this work) are as follows: ~ 1.1 for 4.0 keV·μm^−1^ and ~ 1.45 for 15 keV·μm^−1^ in low (α/β)_x_ tissues, and ~ 1.1 for 4.0 keV·μm^−1^ and ~ 1.35 for 15 keV·μm^−1^ in high (α/β)x tissues. Conversely, standard fractionation schemes (~ 2 Gy (RBE) target doses) can enhance the peak-to-plateau ratio.

Close examination of the R_β_ component for the DDM reveals that for LET of ~ 4 keV·μm^−1^, R_β_ converges to ~ 0.6, while for 15 keV·μm^−1^ R_β_ approaches ~ 1. As described in previous works [13, 14], R_β_ parameterization was obtained by a convenient parameterization which fits the running averages of the experimental data, neglecting any (α/β)_x_ dependencies due to the large uncertainties effecting the β term. Recent works develop a phenomenological model for proton beams from *in vitro* data following a similar approach to R_β_ handling by assuming a negligible (α/β)_x_ dependency [35, 46]. With DDM, parameter fittings are merged to a relatively small amount of data using a running average and thus, this work can shed light on RBE model performance in regions where data is sparse and predictions exhibit large uncertainties. Moreover, existing experimental data is especially scarce for low (α/β)_x_ values (< 3 Gy) [14], where the largest RBE values are expected and the highest variations among the models occur. Further data for low (α/β)_x_ tissues and for clinically-relevant dose levels, especially in standard fractionation regimes (D_RBE_ < ~ 3 Gy (RBE)), are essential for benchmarking the predictive power of these RBE models.

### Experimental benchmarking (in vitro)

RBE model benchmarking through *in vitro* experimentation with a low (α/β)_x_ cell line was the next logical step to verify the significant RBE enhancement observed in the models for dose levels < 4 Gy, a clinically relevant range bearing in mind the typical fractionation size for proton beams of ~ 2 Gy (RBE). Qualitatively, the study investigated both lower LET_d_ (< 10 keV·μm^−1^) and higher LET_d_ (≥10 keV·μm^−1^) values, pertinent endpoints for both normal tissue complication and tumor control probability (TCP). In addition, critical structures surrounding or distal to the target are also associated with the highest LET_d_ values in the study. It is important to note, however, that the *in vitro* data available in the literature is solely based on cell-kill of tumor tissues with RBE as the end point. Therefore, the models provide insight into RBE from the perspective of TCP rather than normal tissue response, which requires the immortalization of normal cell lines to investigate relevant end points [47].

For RBE prediction versus measurement in *experiment A* (Fig. 5), LEM exhibited the highest accuracy for low LET_d_ at dose levels <2Gy, while MKM and DDM performed best for the higher doses. For higher LET_d_ conditions, MKM and DDM both outperformed LEM in predictive power, with local %∆_RBE_ between ~ 1% and ~ 8%, as the dose increases. Although direct comparison of the track-segment condition in silico study shown in Fig. 7 and the monoenergetic beam *in vitro* study is incompatible due to the oversimplification of LET_d_ (neglecting mixed field spectra) and the inherently non-linear relationship of RBE and LET, general trends between the models are consistent.

As for investigations in *experiment B* (clinical-like fields in Fig. 6), interpretation becomes more convoluted when considering the complex mixed radiation field. In general, DDM and MKM demonstrated the lowest local |%∆_RBE_| of < 10%, overall. As anticipated, |%∆_RBE_| decreased with increasing dose for all three models. Disagreement in the lower LET_d_ condition can be explained by the scarce amount of data for low LET_d_, especially with cell lines with (α/β)_x_ < 3 Gy, which suggests that further *in vitro* study and tweaking of the models could yield improved RBE predictions. Nevertheless, 5% to 10% predictive power for RBE in the target region is acceptable considering the uncertainty of the reference photon sensitivity measurement. For the entrance channel condition in Fig. 6, all three models (especially DDM) tend to overestimate RBE for < 1 Gy, a typical fractionation treatment dose range, offering a conservative estimate for normal tissue in the plateau region.

DDM depends only on the (α/β)_x_ ratio while the MKM, instead, depends also on the absolute value of β_x_, which contributes in the determination of z_sat_ [22]. Low β_x_ values result in a reduced saturation coefficient, leading to RBE enhancement. To further shed light on this point, calculations were performed with the two fields arrangement applying (α/β)_x_ = 2.0 Gy, planned D_RBE_ = 4 Gy (data not shown) and β_x_ = 0.02 Gy^− 2^, finding consistently higher D_RBE_ values (about 8%). In contrast, LEM depends on multiple parameters, including α_x_, β_x_ and D_t_. By varying α_x_ and β_x_ by 25% but maintaining the same (α/β)_x_, no measurable dependence of RBE_α_ was found for clinically-relevant LET values using carbon ion beams, with a limited effect on the RBE at 10% survival [48].

### Clinical outlook

Regarding patient dose calculation, LET_d_ prediction for the prostate case was in line with the findings from the SOBP study; however, the head case plan exhibited lower LET_d_ values since the energy spread of the beam is increased by the ripple filter (RiFi) to reduce BP sharpness for clinically acceptable target dose homogeneity. Furthermore, FRoG calculated physical and biological dose distributions were in good agreement with FLUKA MC and well within clinically acceptable tolerances. At HIT, both the MCTP and FRoG dose engine are functional for helium ion beam therapy, enabling future treatment planning comparison and robust RBE optimization studies necessary before and during clinical trials, as performed in previous works for carbon ions [49]. In addition, the FRoG platform will support the development and validation of the first analytical TPS for helium ion beams, providing multiple biological models for clinical research.

As HIT prepares for clinical translation of ^4^He, the findings and efforts in this work may serve as a starting point for clinical decision making. Currently, there is no official consensus as to which RBE model for helium ions is best suited for treatment and whether a single tissue approximation for biological dose prediction will be used as done with carbon ions. In light of these issues, the FRoG platform includes all three models presented in this work, as well as tissue-dependent biological dose calculation, providing valuable insight into radiological uncertainty during treatment planning. Regarding optimization of a next generation TPS for particle therapy, advanced optimization strategies are recommended considering the large uncertainties associated with biological modeling and the lack of evidence supporting *in vitro* model applicability to *in vivo* settings [50]. With technqiues like RBE/LET gradient minimization in the target, constant over- or under-estimation of D_RBE_ could be detected in an initial dose-escalation phase. At HIT, a systematic clinical investigation with an initial group of patients is anticipated to observe and analyze clinical outcome.

All presented RBE models are based on the same set (or sub-set) of the published biological *in vitro* data, used repeatedly for model tuning and benchmarking purposes. *In vivo* data is sparse at best and rarely used to verify the models’ predictions [51]. The experimental and intrinsic uncertainties in the data constrain the confidence in these models to a degree which is less than clinically desirable, yielding model fits with significant variation. It is worth noting here that the agreement of the LEM used for this study with respect to the other models might further improve if the same set of *in vitro* data would have been used for tuning the LEM, as done for the DDM and MKM. These findings suggest that systematics in RBE predictions in the high dose region for clinical ^4^He ion treatment fields due to different choices of RBE modelling approaches can be restricted to be mostly within 10% to 15% when tuning the parameters of the RBE models to the same (or a similar) set of the available *in vitro* cell data for ^4^He ions.

In turn, this may imply that systematic uncertainties in the prediction of RBE for helium ions for clinical scenarios are not primarily dominated by the choice of the RBE model, but instead dictated by the choice of the *in vitro* dataset and methodology used for tuning the RBE model parameters. Similar conclusions might hold true for RBE models of higher Z ion species. Additional systematic RBE uncertainties arise from differences between *in vivo* and *in vitro* data; however, due to their scarcity, *in vivo* and clinical data are hardly used to tune RBE models, but rather for validation of commonly established RBE models [52], exception being the neutron-equivalent scaling point used for carbon ions [53, 54]. Previous works also propose application of clinical data for RBE model tuning in addition to *in vitro* and *in vivo* measurements [55].

For helium ions, it is certainly challenging to make definitive statements about RBE considering the lacking of experimental data. To reduce RBE model uncertainties for ^4^He, collecting additional evidence, especially *in vivo*, is recommended before clinical application. However, the differences in RBE predictions found in this study for the three presented models are similar to the RBE variation for *in vitro* data in proton beams, which are typically knowingly accepted when assuming RBE = 1.1 [47]. Ultimately, the choice of model and tissue type for biological dose optimization is a clinical decision to ensure the most safe and effective patient treatment and care possible.

## Conclusion

Before the start-up of a ^4^He ion beam therapy program, a comprehensive evaluation of the variable RBE and the associated models is critical. The main dependencies of three RBE models for ^4^He ion beam therapy were studied in silico and validated against *in vitro* experimentation with a radio-resistant tumor cell line. Clinically relevant uncertainties were observed, especially for low (α/β)_x_ values where the available literature data are scarce. The observed uncertainties between the models as well as variability of RBE as a function of its dependency (especially for low (α/β)_x_ tissues commonly treated with particle therapy) suggest that the selection, refinement and validation of either a biophysical/mechanistic- or phenomenological-based approach are essential prior to clinical translation of helium ion beam therapy.

## Additional file

Additional file 1: Supplementary data analysis for biological dose prediction using ^4^He ions, including SOBPs for a parallel opposed beam plan (two-field), DVH statistics for FRoG against FLUKA MC for the two patient cases, and EUD calculations comparing the three investigated RBE models. (DOCX 1421 kb)

## Abbreviations

BP: Bragg peak
CT: Computed tomography
DDM: Data-driven model
DVH: Dose volume histogram
EUD: Equivalent uniform dose
HIT: Heidelberg Ion-beam Therapy Center
LEM: Local effect model
LET: Linear energy transfer
MC: Monte Carlo
MCTP: Monte Carlo treatment planning platform
MKM: Microdosimetric kinetic model
OAR: Organ at risk
PTV: Planning target volume
RBE: Relative biological effectiveness
SOBP: Spread-out Bragg peak
TPS: Treatment planning system
