# Supplementary material for: Biophysical modeling and experimental validation of relative biological effectiveness (RBE) for 4He ion beam therapy
Source: Radiat Oncol. 2019 Jul 11;14:123. doi: 10.1186/s13014-019-1295-z (PMC6624994; doi:10.1186/s13014-019-1295-z)
Supplement: Supplementary file 1 — Supplementary data analysis for biological dose prediction using 4He ions, including SOBPs for a parallel opposed beam plan (two-field), DVH statistics for FRoG against FLUKA MC for the two patient cases, and EUD calculations comparing the three investigated RBE models. (DOCX 1421 kb) [file 13014_2019_1295_MOESM1_ESM.docx]

**
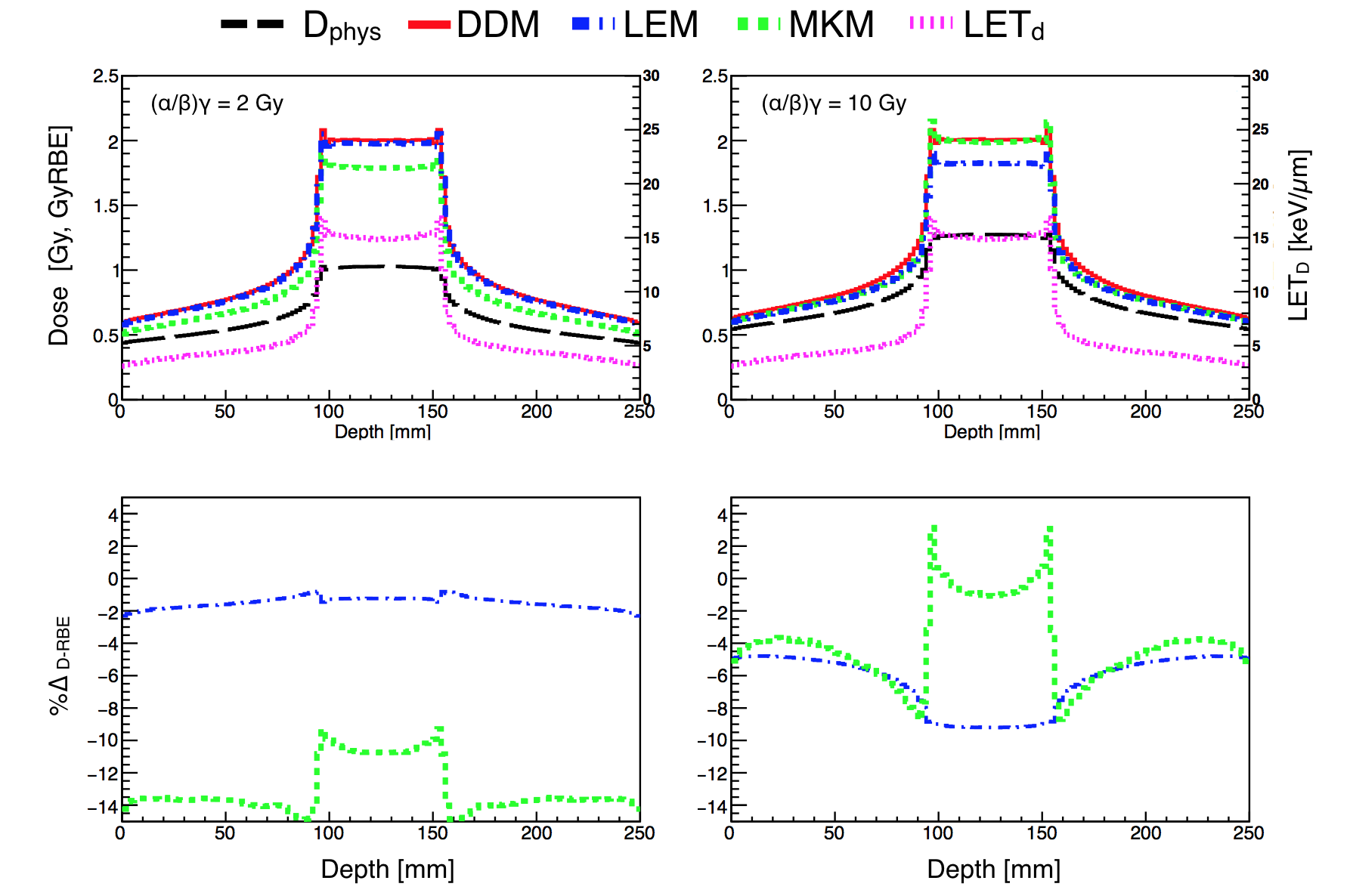
**

**Supplementary figure 1.** SOBPs at 2 Gy (RBE) dose level with two opposing beam arrangements in water, optimized using the DDM and assuming (α/β)_x_ = 2 Gy (upper left) and (α/β)_x_ = 10 Gy (upper right) are plotted against forward calculations applying LEM and MKM. The total dose (D_phys_) and LET_d_ distributions as function of the depth in water are also reported. The lower panels present %∆_D-RBE_, between planned and forward calculated D_RBE_ for MKM and LEM as function of depth in water.

**Supplementary table 1.** DVH Statistics for the validation of FRoG against FLUKA MC. Physical dose and RBE-weighted doses (DDM, LEM and MKM) are provided with percent difference from the reference (%∆).

**
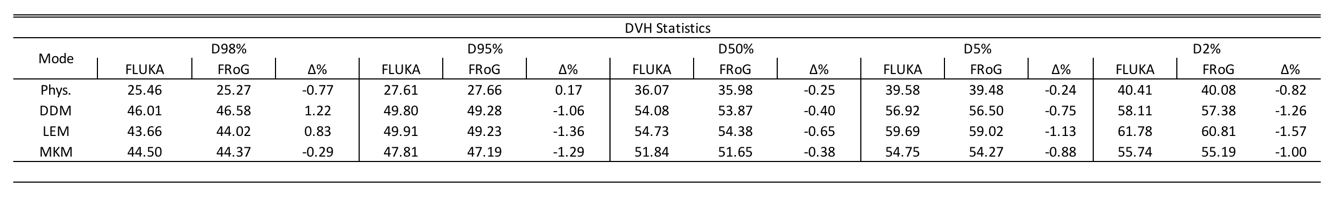
**

**Supplementary table 2.** Percentage variation of the calculated EUD (∆EUD) between the three biological models and the data-driven approach for all the configurations in water studied with (α/β)_x_ = 2 Gy.

**Supplementary table 3.** Percentage variation of the calculated EUD (∆EUD) between the three biological models and the data-driven approach for all the configurations in water studied with (α/β)_x_ = 10 Gy.
